# Long non-coding RNA NEAT1 promotes angiogenesis in hepatoma carcinoma via the miR-125a-5p/VEGF pathway

**DOI:** 10.1515/biol-2022-0498

**Published:** 2022-09-19

**Authors:** Jingyun Guo, Qi Yuan, Yuan Fang, Jinmao Liao, Zheng Zhang

**Affiliations:** Department of Hepatopathy, The Hunan Provincial People’s Hospital, The First Affiliated Hospital of Hunan Normal University, No. 61, Jiefang Road West, Changsha, Hunan, 410005, China

**Keywords:** NEAT1, miR-125a-5p, angiogenesis, VEGF, hepatoma carcinoma

## Abstract

The study’s purpose was to investigate the biological function of long non-coding RNA nuclear paraspeckle assembly transcript 1 (NEAT1) in hepatoma carcinoma (HCC). HCC tissues and cells exhibited increased levels of NEAT1 and decreased levels of miR-125a-5p. Reduction in the expression of NEAT suppressed HepG2 cell proliferation and increased apoptosis. This was accompanied by suppression of the AKT/mTOR and ERK pathways, while the opposite was observed for miR-125a-5p. Angiogenesis assay results indicated that NEAT was proangiogenic. A dual-luciferase reporter assay indicated that NEAT1 was bound to miR-125a-5p and miR-125a-5p was bound to vascular endothelial growth factor (VEGF). The proangiogenic effects of NEAT and its stimulation of AKT/mTOR and ERK were reversed by miR-125a-5p. The anti-angiogenic effects of miR-125a-5p and its inhibitory effect on AKT/mTOR and ERK pathways were reversed by co-incubation with VEGF. The conclusion was that NEAT1 enhances angiogenesis in HCC by VEGF via a competing endogenous RNA (ceRNA) of miR-125a-5p that regulates AKT/mTOR and ERK pathways.

## 1 Introduction

Hepatic carcinoma (HCC) is a primary malignant tumor with relatively high morbidity and mortality [1,2]. HCC progression is a complicated process involving abnormal activation of various cellular and molecular patterns and an imbalance between oncogenes and tumor suppressor genes [3]. In recent years, molecular targeting therapy has been applied clinically to treat malignant tumors, including HCC, exerting antitumor effects by interfering with the expression of carcinogenic proteins and RNAs [4,5]. In addition, owing to rapid disease progression, it is currently difficult to diagnose HCC at an early stage. Most patients are diagnosed with HCC that has already reached an advanced stage [6,7]. Thus, there is a pressing urgency to explore novel molecular markers for the diagnosis and therapy of clinical HCC.

Angiogenesis is closely correlated with HCC progression [8], as tumor growth is dependent on angiogenesis [9]. The proliferation of tumor cells is significantly promoted by the absorption of nutrition from blood vessels [10]. Therefore, angiogenesis is a key step in tumor growth, metastasis, and infiltration. Tumor angiogenesis is regulated by multiple factors, among which vascular endothelial growth factor (VEGF) is currently recognized as a specific angiogenesis-promoting factor. VEGF is overexpressed in almost all malignant tumors [11]. VEGF inhibitors are currently used as first-line treatment for advanced HCC [12]. Therefore, investigation of VEGF targeting in HCC has ample clinical relevance.

Long non-coding RNAs (lncRNAs) play crucial roles as biomarkers and therapeutic targets in HCC [13]. Competing endogenous RNA (ceRNA) mechanisms have been extensively studied in HCC [14]. For example, lncRNA MYLK-AS1 promotes angiogenesis by regulating the miR-424-5p/E2F7/VEGFR-2 pathway [15]. LINC00662 promotes HCC progression via the Wnt/β-catenin pathway [16]. Nuclear paraspeckle assembly transcript 1 (NEAT1) is enriched in the nucleus and drives multiple cancers, including HCC, breast cancer, and colorectal cancer [17,18,19,20,21]. NEAT1 promotes HCC proliferation via miR-320a/L antigen family member 3 [21,22]. NEAT1 regulates HCC cell proliferation, apoptosis, and invasion via the miR-199a-3p/UCK2 pathway [18,23,24,25]. Recently, microRNAs (miRNAs) have been reported to play a key role in angiogenesis in HCC. miR-325-3p drives angiogenesis in HCC via the CXCL17/CXCR8 pathway [26]. miR-125a-5p, a tumor suppressor [27,28], has been reported to inhibit tumorigenesis by targeting VEGF in HCC [29]. However, the potential effects and mechanism of NEAT1 in terms of miR-125a-5p involvement in HCC have not been fully elucidated.

In the present study, the effects of NEAT1 on angiogenesis in HCC were investigated to determine its potential application as a tumor suppressor in the diagnosis and treatment of clinical HCC.

## 2 Materials and methods

### 2.1 HCC tissues and cell lines

Three pairs of HCC and para-carcinoma tissues were isolated from HCC patients in the Hunan Provincial People’s Hospital. Normal hepatocyte LO2 cells, the HCC cell lines (HepG2, SMMC-7721, and MHCC97H), and HUVEC were obtained from the Institute of Biochemistry and Cell Biology of China.


**Informed consent:** Informed consent has been obtained from all individuals included in this study.
**Ethical approval:** The research related to human use has been complied with all the relevant national regulations, institutional policies and in accordance with the tenets of the Helsinki Declaration, and has been approved by the Hunan Provincial People’s Hospital.

### 2.2 Treatment of the cells

The HepG2/HUVEC cells were divided into five groups, including a control, a negative inhibitor (NC) control, a NEAT1 inhibitor, a mimic NC, and a NEAT1 mimic group, as well as a miR-125a-5p treatment group. HepG2 cells and HUVEC were transfected with 100 nM NEAT1/miR-125a-5p mimic, mimic NC, NEAT1/miR-125a-5p inhibitor, or NC inhibitor using Lipofectamine 3000 for 48 h. The transfection efficacy was evaluated by qRT-PCR. The cells in the VEGF group were treated with 50 ng/mL VEGF (cat#ab9571, Abcam) for 24 h.

### 2.3 qRT-PCR

RNA was extracted from the tissues or cells using TRIzol reagent (Thermo Fisher Scientific) and mixed with PrimeScript kit reagents to generate cDNA. A qPCR SuperMix kit (Thermo Fisher Scientific) was used to amplify the PCR, and the 2^−ΔΔCt^ method was used to determine the relative expression levels of genes. The primer sequences used were as follows, lnc NEAT1, sense 5′-AAACGCTGGGAGGGTACAAG-3′, antisense 5′-ATGCCCAAACTAGACCTGCC-3′; β-actin, sense 5′-ACCCTGAAGTACCCCATCGAG-3′, antisense 5′-AGCACAGCCTGGATAGCAAC-3′; miR-125a-5p, sense 5′-CCTGAGACCCTTTAACCTGTGAA-3′; U6, sense 5′-CTCGCTTCGGCAGCACA-3′, antisense 5′-AACGCTTCACGAATTTGCGT-3′.

### 2.4 Western blot analysis

Following lysis of the cells, the lysates were subjected to 15% SDS-PAGE and the proteins transferred to PVDF membranes (Thermo Fisher Scientific) by semi-dry transfer. The membranes were blocked with 5–10% BSA solution for 1.5 h and then incubated with specific antibodies for 2 h. After washing, the membranes were incubated for 2 h at 25°C with a horseradish peroxidase-conjugated secondary antibody. Details regarding the antibodies used are provided in Table 1. Antigens visualized using a chemiluminescence imaging system (Guangzhou Qinxiang, China). Quantity One software was used to analyze the grey-scale values. β-actin was used as an internal reference. The signal intensities are presented as the corresponding protein antigen/β-actin levels.

**Table 1:** The antibody information

Index name	Cat. no.	Dilution	Manufacturer	Production address
VEGFA	66828-1-Ig	1:1,000	Proteintech	USA
AKT	10176-2-AP	1:1,000	Proteintech	USA
p-AKT	66444-1-Ig	1:3,000	Proteintech	USA
mTOR	#2972	1:1,000	CST	USA
p-mTOR	#5536	1:1,000	CST	USA
ERK1/2	ab184699	1:10,000	Abcam	UK
p-ERK1/2	4370S	1:1,000	CST	USA
Cleaved Caspase 3	#9661	1:1,000	CST	USA
Bcl-2	ab182858	1:2,000	Abcam	UK
β-actin	66009-1-Ig	1:5,000	Proteintech	USA
HRP goat anti-mouse IgG	SA00001-1	1:5,000	Proteintech	USA
HRP goat anti-rabbit IgG	SA00001-2	1:6,000	Proteintech	USA

### 2.5 Cell counting kit-8 (CCK-8) assay

Cell proliferation was measured using a CCK-8 assay (Sigma-Aldrich). After being seeded into a 96-well plate, the cells were incubated for 24 h at 37°C. Different reagents were added to the plate and incubated for 24 h. Next 10 μL of CCK-8 solution was added and the cells were incubated for 2 h. The optical density (OD) of the solution was measured using a Benchmark™ microplate reader (Bio-Rad, CA, USA) at 450 nm.

### 2.6 Flow cytometry

The cells were seeded into plates and incubated with 10 μL of fluorescently labeled Annexin V reagent. After adding 6 μL of Propidium Iodide (PI) solution and incubation for 15 min, the cells were transferred to a tube with PBS and assayed by flow cytometry (BD).

### 2.7 Angiogenesis assay

HUVEC (1 × 10^4^) were placed in the wells of a Matrigel (BD)-coated µ-Slide angiogenesis chamber to assay tube formation. Following incubation for 6 h, light microscopy images were taken and ImageJ software was used to determine the total tube length.

### 2.8 Dual-luciferase reporter assay

Wild-type (WT) and mutant NEAT1/VEGF-3’-UTR sequences (NEAT1/VEGF-WT and NEAT1/VEGF-MUT, respectively) were cloned into pGL3 luciferase reporter plasmid (Thermo Fisher Scientific) to generate pGL3-NEAT1/VEGF-WT and pGL3-NEAT1/VEGF-MUT, respectively. HepG2 cells (4.5 × 10^5^ cells/well) were co-transfected with either pGL3-NEAT1/VEGF-WT or pGL3-NEAT1/VEGF-MUT and either miR-125a-5p mimic or an empty vector using Lipofectamine 3000 (Invitrogen).

### 2.9 Statistical analysis

The data are presented as mean values ± the standard deviation (SD) and analyzed with GraphPad software. Student’s *t*-test and one-way analysis of variance (ANOVA) were used to compare differences among the various groups. *p* < 0.05 was considered statistically significant.

## 3 Results

### 3.1 Increased NEAT1 and decreased miR-125a-5p expression in HCC tissues

**Figure 1:**
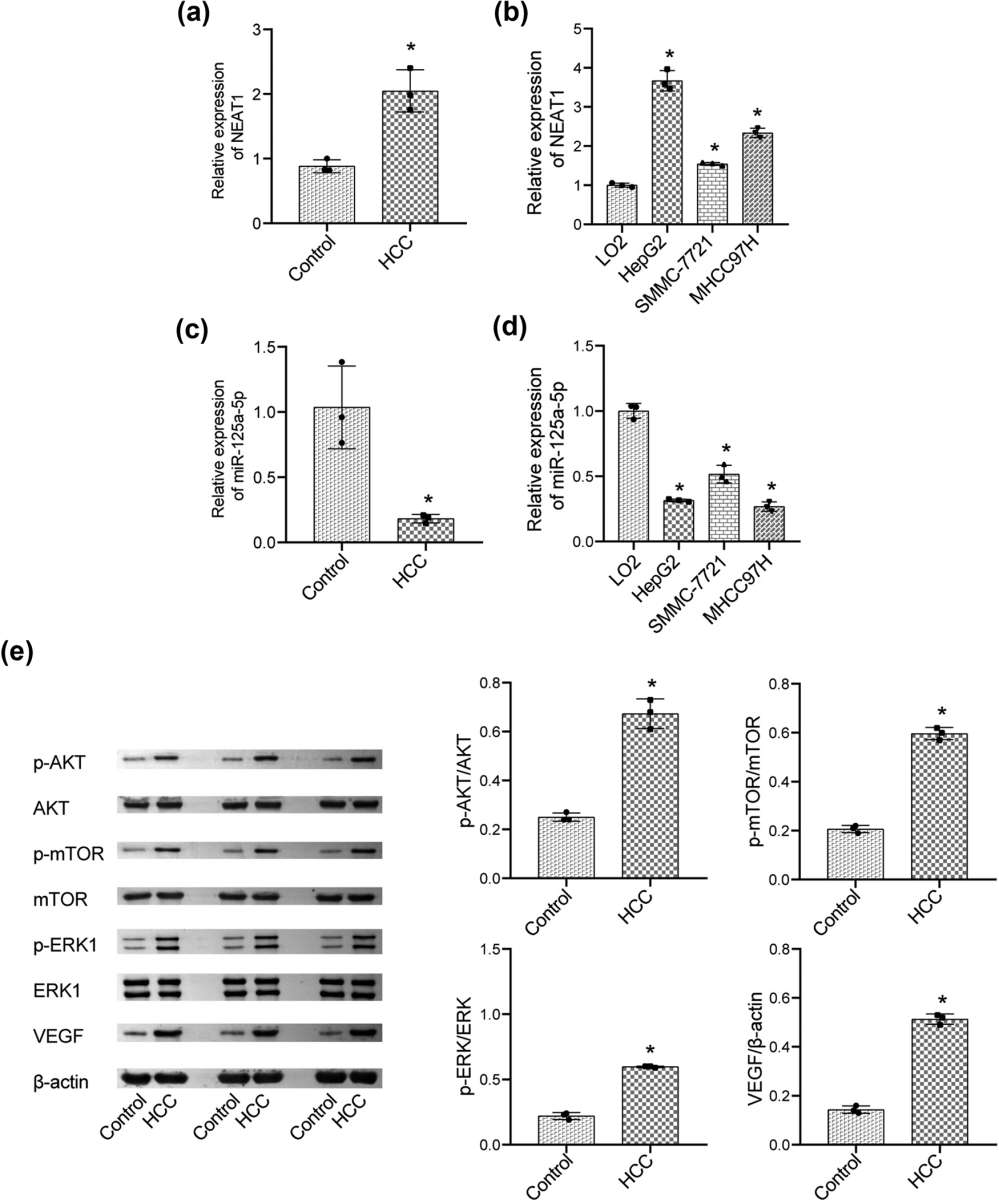
Increased NEAT1 and decreased miR-125a-5p expression in HCC tissues. (a and b) qRT-PCR was utilized to determine the expression level of NEAT1 in HCC tissues and cells. (c and d) qRT-PCR was utilized to determine the expression level of miR-125a-5p in HCC tissues and cells. (e) The level of AKT, p-AKT, mTOR, p-mTOR, ERK1/2, p-ERK1/2, and VEGF was evaluated by western blot (*p < 0.05 vs control).

As shown in Figure 1a, compared to the control, NEAT1 expression was elevated in HCC tissues. Figure 1b shows the expression level of NEAT1 in normal hepatocytes (LO2) and HCC cell lines (HepG2, SMMC-7720, and MHCC97H). We found that, compared to LO2 cells, the expression of NEAT1 was greatly increased in HCC cell lines, of which HepG2 cells expressed the highest level. The qRT-PCR results revealed that the expression level of miR-125a-5p was significantly downregulated in HCC (Figure 1c and d). Therefore, HepG2 cells were used in all subsequent experiments. To detect activation of VEGF and the downstream pathway, the levels of p-AKT, AKT, p-mTOR, mTOR, p-ERK1/2, ERK1/2, and VEGF were assayed in both HCC and para-carcinoma tissues (control). As shown in Figure 1e, in contrast to the control, p-AKT, p-mTOR, p-ERK1/2, and VEGF levels were significantly upregulated in HCC tissues, with the level of AKT, mTOR, and ERK1/2 unchanged, indicating that phosphorylation of the AKT/mTOR/ERK pathway components was increased.

### 3.2 NEAT1 promotes HepG2 cell proliferation by AKT/mTOR/ERK/VEGF signaling

**Figure 2:**
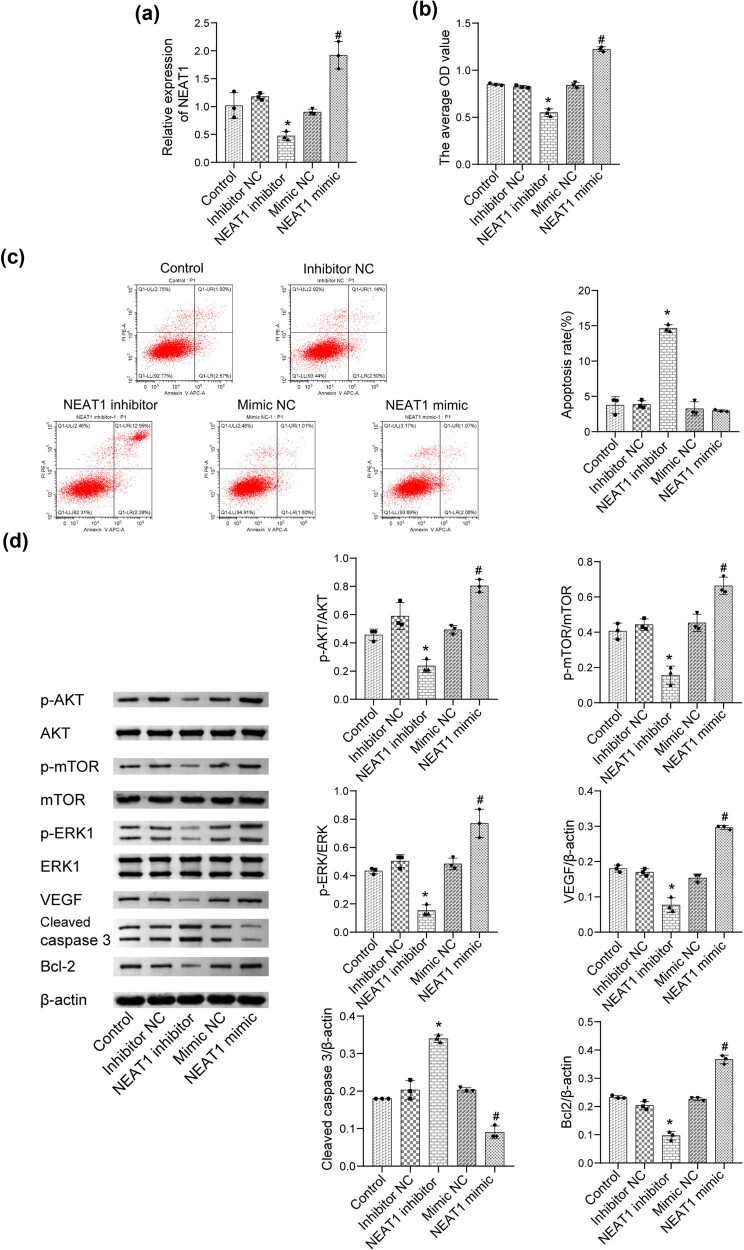
NEAT1 promotes HepG2 cell proliferation by AKT/mTOR/ERK/VEGF signaling. (a) The expression level of NEAT1 in HepG2 cells was determined by qRT-PCR. (b) CCK-8 assay was used to measure the proliferation of HepG2 cells. (c) The level of apoptosis of HepG2 cells was assayed by flow cytometry. (d) The levels of AKT, p-AKT, mTOR, p-mTOR, ERK1/2, p-ERK1/2, VEGF, cleaved caspase-3, and Bcl-2 were evaluated by western blot (*p < 0.05 vs inhibitor NC; ^#^ *p* < 0.05 vs mimic NC).

As shown in Figure 2a, compared with the control, NEAT1 was significantly downregulated in the NEAT1 inhibitor group and upregulated in the NEAT1 mimic group, indicating that NEAT1 overexpression or knockdown in HepG2 cells was established successfully. As shown in Figure 2b, cell proliferation was significantly suppressed in the NEAT1 inhibitor group and was promoted in the NEAT1 mimic group. As shown in Figure 2c, the apoptosis rate in the NEAT1 inhibitor group was higher than that in the NC inhibitor group. As shown in Figure 2d, the levels of p-AKT, p-mTOR, p-ERK1/2, and VEGF were significantly reduced in the NEAT1 inhibitor group and increased in the NEAT1 mimic group, compared to those in the NC group. In addition, the level of Bcl-2 was reduced and that of cleaved caspase-3 was elevated in the NEAT1 inhibitor group. Opposite effects were observed in the NEAT1 mimic group. Altogether, these results show that NEAT1 promotes HepG2 cell proliferation by AKT/mTOR/ERK/VEGF pathway.

### 3.3 miR-125a-5p inhibits cell proliferation by targeting AKT/mTOR/ERK/VEGF signaling

**Figure 3:**
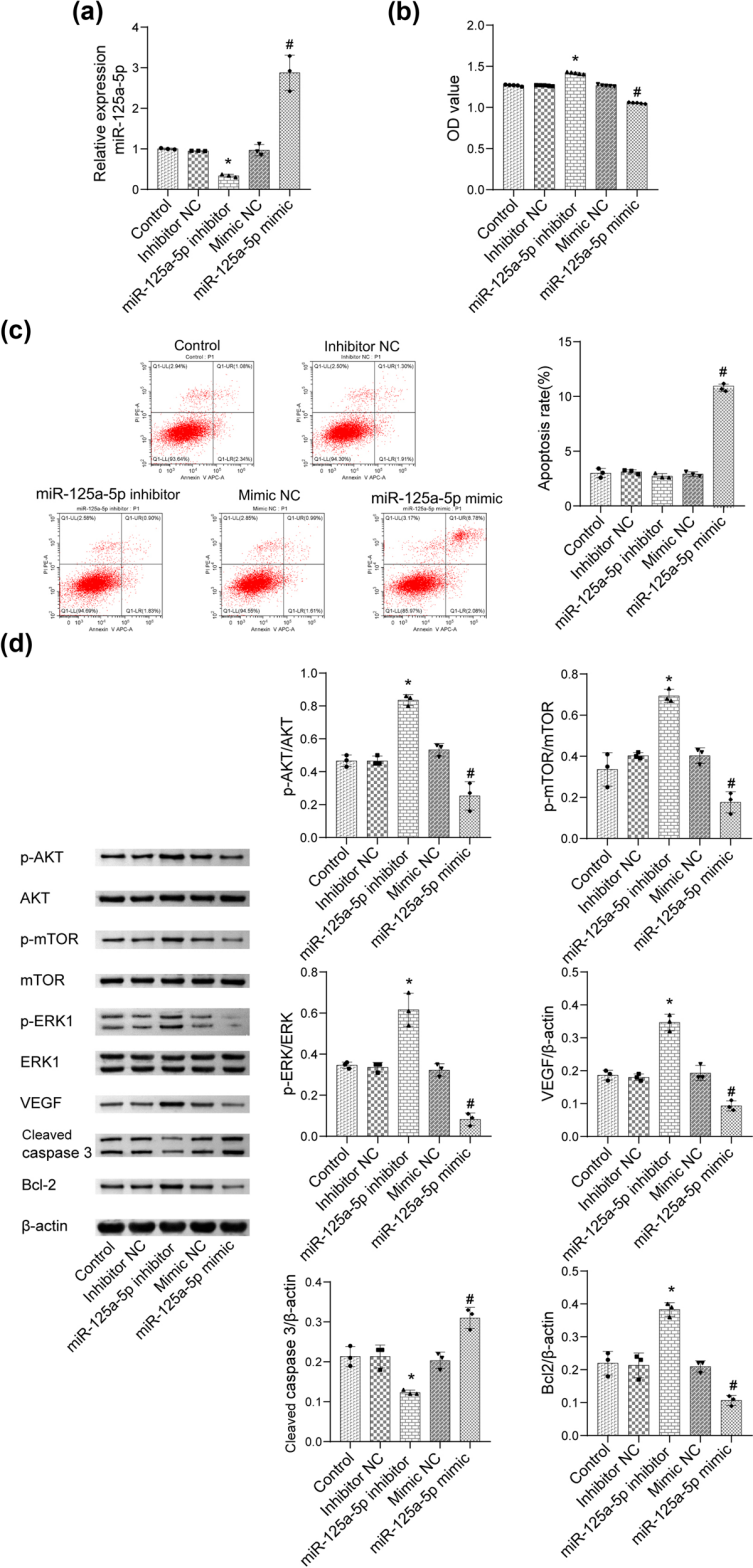
Cell proliferation can be inhibited by miR-125a-5p/AKT/mTOR/ERK pathway. (a) The expression level of miR-125a-5p in HepG2 cells was determined by qRT-PCR. (b) CCK-8 assay was used to measure the proliferation of HepG2 cells. (c) The level of apoptosis of HepG2 cells was assayed by flow cytometry. (d) The levels of AKT, p-AKT, mTOR, p-mTOR, ERK1/2, p-ERK1/2, VEGF, cleaved caspase-3, and Bcl-2 were evaluated by western blot (*p < 0.05 vs inhibitor NC; ^#^ *p* < 0.05 vs mimic NC).

As shown in Figure 3a, the qRT-PCR results revealed that the expression level of miR-125a-5p was significantly suppressed in the miR-125a-5p inhibitor group and enhanced in the miR-125a-5p mimic group. The OD value was upregulated in the miR-125a-5p inhibitor group and downregulated in the miR-125a-5p mimic group (Figure 3b). As shown in Figure 3c, the apoptosis rate in the miR-125a-5p mimic group was higher than that in the mimic-NC group. Figure 3d shows that compared to the NC group, the levels of p-AKT, p-mTOR, p-ERK1/2, VEGF, and Bcl-2 were significantly increased in the miR-125a-5p inhibitor group and inhibited in the miR-125a-5p mimic group. The level of cleaved caspase-3 was reduced in the miR-125a-5p inhibitor group. The opposite effects were observed in the miR-125a-5p mimic group. These results illustrate that miR-125a-5p inhibits cell proliferation by targeting AKT/mTOR/ERK/VEGF pathway.

### 3.4 NEAT1 can enhance angiogenesis via miR-125a-5p

**Figure 4:**
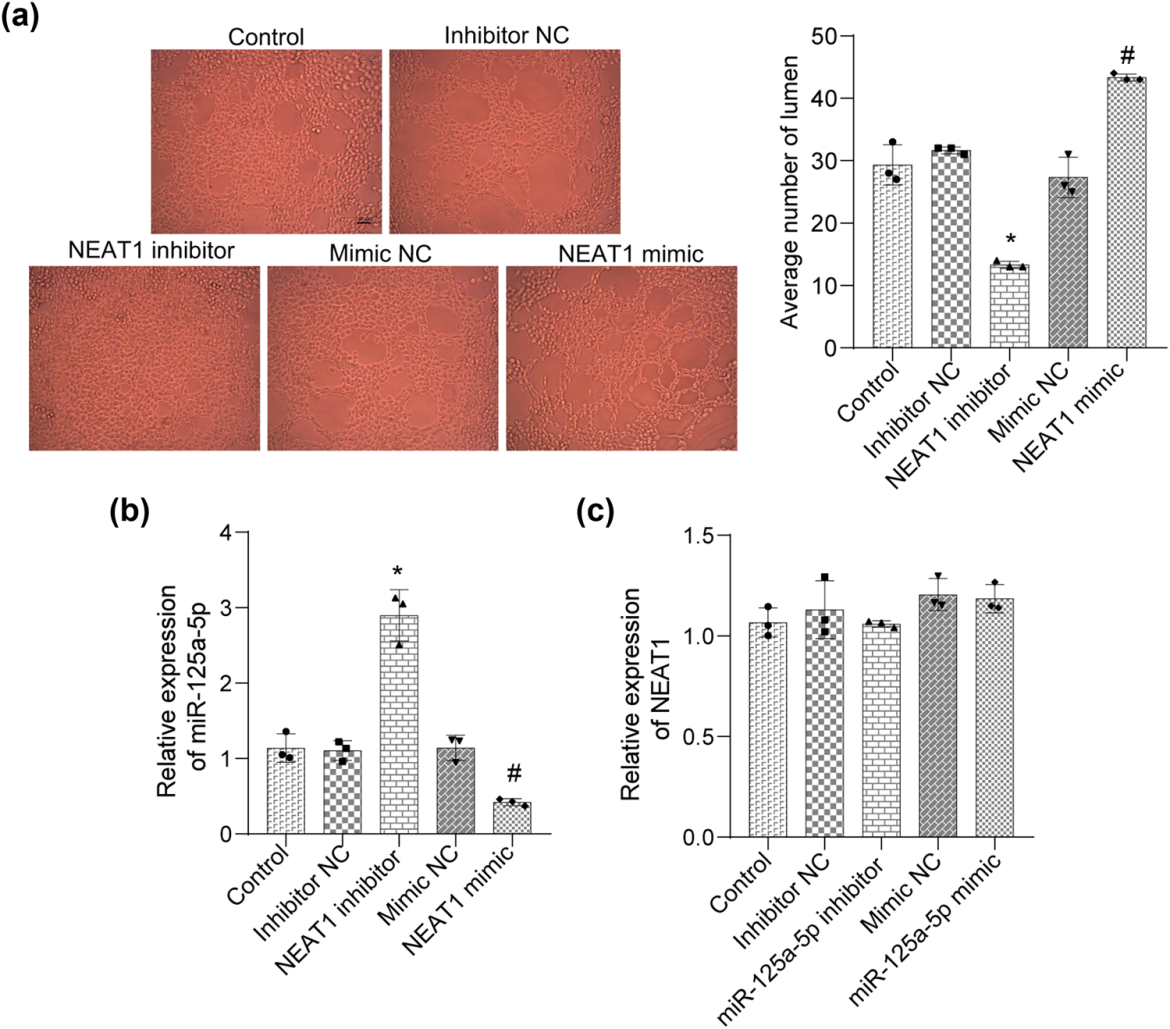
NEAT1 may enhance angiogenesis. (a) An angiogenesis assay was utilized to assay angiogenesis with different HUVEC treatments (scale bars = 100 µm). (b and c) The expression level of miR-125a-5p and NEAT1 in HepG2 cells was determined by qRT-PCR. (*p < 0.05 vs inhibitor NC; ^#^ *p* < 0.05 vs mimic NC).

To further study the effects of NEAT1 on angiogenesis, we performed an angiogenesis assay. The results showed that the average number of lumens was suppressed in the NEAT1 inhibitor group and increased in the NEAT1 mimic group (Figure 4a). The results of the qRT-PCR experiment showed that the NEAT1 inhibitor promoted the expression of miR-125a-5p, whereas the NEAT1 mimic inhibited the expression of miR-125a-5p, suggesting that NEAT1 can negatively regulate the expression of miR-125a-5p (Figure 4b). We further investigated the effects of miR-125a-5p on NEAT1 expression. The results showed that interference or overexpression of miR-125a-5p had no significant effect on NEAT1 expression (Figure 4c). These results suggest that NEAT1 may enhance angiogenesis via miR-125a-5p.

### 3.5 NEAT1 can enhance angiogenesis by acting as a competing ceRNA of miR-125a-5p

**Figure 5:**
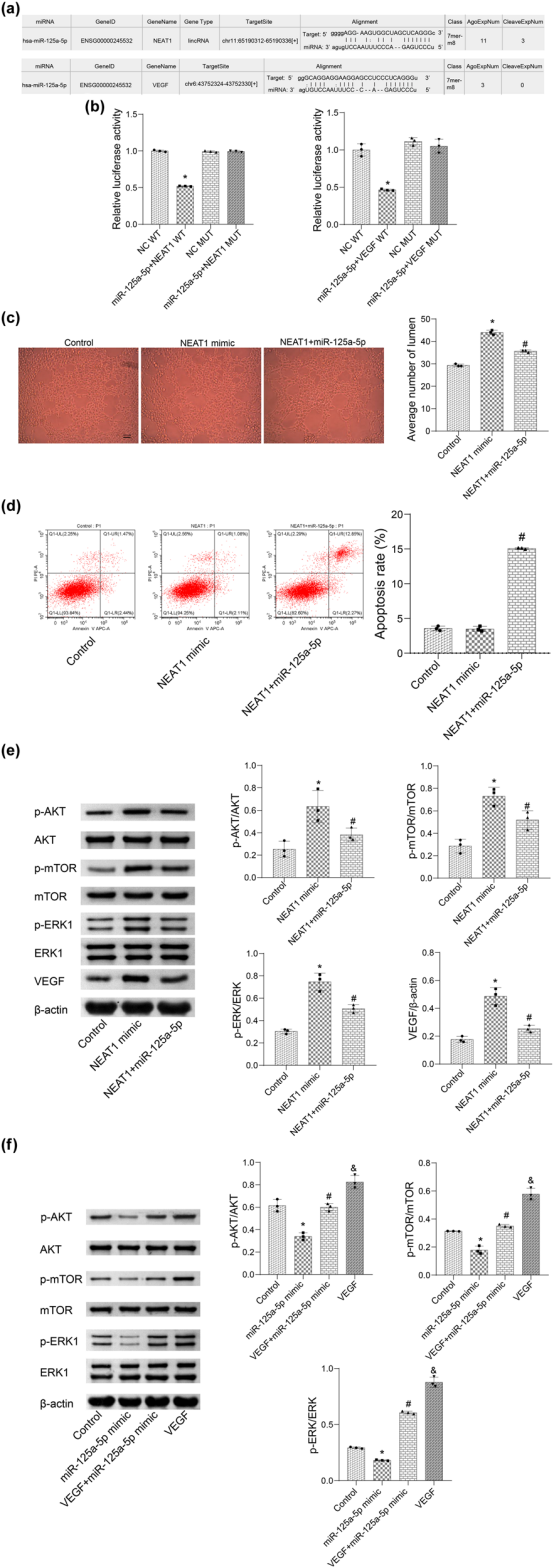
NEAT1 may enhance angiogenesis by acting as a ceRNA of miR-125a-5p. (a and b) A dual-luciferase reporter assay evaluated the binding correlation between NEAT1 and miR-125a-5p (*p < 0.05 vs NC WT) as well as the binding correlation between miR-125a-5p and VEGF (*p < 0.05 vs NC WT). (c and d) Angiogenesis and flow cytometry assays were used to assess angiogenesis and apoptosis, respectively. (e) The levels of p-AKT, AKT, p-mTOR, mTOR, p-ERK1/2, ERK1/2, and VEGF were determined by western blot (*p < 0.05 vs control, ^#^ *p* < 0.05 vs NEAT1). (f) The levels of p-AKT, AKT, p-mTOR, mTOR, p-ERK1/2, and ERK1/2 were determined by Western blot (*p < 0.05 vs control, ^#^ *p* < 0.05 vs miR-125a-5p mimic, ^&^ *p* < 0.05 vs VEGF + miR-125a-5p mimic).

To study the potential RNA target of miR-125a-5p, we used the online bioinformatics database StarBase 3.0 (http://starbase.sysu.edu.cn/). The potential binding sites are shown in Figure 5a. A dual-luciferase reporter assay was performed to verify the relationship between NEAT1, miR-125a-5p, and VEGF. As shown in Figure 5b, the relative luciferase activity decreased significantly in cells transfected with a miR-125a-5p mimic in the presence of NEAT1 or VEGF 3′-UTR WT compared to that in cells transfected with mimic NC. However, there were no significant differences in the fluorescence intensity in the cells transfected with the miR-125a-5p mimic and mimic NC in the presence of NEAT1 or VEGF 3′-UTR MUT. These results indicate that NEAT1 can target miR-125a-5p and miR-125a-5p can target VEGF. As shown in Figure 5c, compared to the control, the average number of lumens was increased in the NEAT1 mimic group, and this was reversed by miR-125a-5p. Simultaneous overexpression of NEAT1 and miR-125a-5p promoted cell apoptosis compared with the NEAT1 mimic group (Figure 5d). To explore possible reasons for the effects of NEAT1, we measured the levels of p-AKT/AKT, p-mTOR/mTOR, p-ERK/ERK, and VEGF. The results demonstrated that NEAT1 activated AKT/mTOR/ERK/VEGF pathway, while the effects were reversed by miR-125a-5p (Figure 5e). We further evaluated the activation state of the AKT/mTOR/ERK pathway in each group. As shown in Figure 5f, compared with the control, significant reductions in the levels of p-AKT/AKT, p-mTOR, mTOR, and p-ERK/ERK were observed in the miR-125a-5p mimic group, which was greatly elevated by the introduction of VEGF. Compared with the miR-125a-5p mimic and VEGF groups, the levels of p-AKT, p-mTOR, and p-ERK1/2 were significantly reversed in the VEGF + miR-125a-5p group. Thus, NEAT1 may enhance angiogenesis by acting as a ceRNA of miR-125a-5p and affect AKT/mTOR/ERK/VEGF pathway.

## 4 Discussion

The mechanisms underlying tumor angiogenesis has been widely investigated, and significant achievements have been made based on this theory [30]. Angiogenesis is an important biological process in the formation and progression of HCC, which is a well-known type of solid tumor [31]. Antiangiogenic treatments have already been reported to exert promising antitumor activities against HCC by inhibiting formation of the vasculature and nutrition supply [32]. Several antiangiogenic drugs are in clinical use, including sorafenib, regorafenib, lenvatinib, and cabozantinib [33]. However, the safety and efficacy of antiangiogenic therapies remain controversial. In recent years, miRNAs have been reported to regulate angiogenesis [34]. miRNAs can mediate the function of endothelial cells in both non-autonomous and autonomous manner during tumor angiogenesis, and the expression levels of pro- or anti-angiogenic factors in tumor cells can be regulated by miRNAs [34]. miR-20, miR-200, miR-29, and miR-497 target angiogenic genes, thereby either inhibiting or promoting endothelial cell angiogenesis [35]. We found that the expression level of miR-125a-5p was greatly reduced in HCC. We further established a miR-125a-5p overexpression and knockdown model in HepG2 cells. Proliferation of these cells was inhibited by miR-125a-5p overexpression, indicating a promising inhibitory effect of miR-125a-5p against HCC progression. In addition, the results of the angiogenesis assay indicated that angiogenesis in HUVEC was significantly inhibited by miR-125a-5p. The relationship between miR-125a-5p and VEGF expression was examined using a dual-luciferase reporter assay. Upon co-incubation with VEGF, the anti-angiogenic effect of miR-125a-5p was reversed obviously, indicating that the inhibitory effect of miR-125a-5p on angiogenesis was related to downregulation of VEGF. In our future work, the antitumor efficacy of miR-125a-5p will be investigated further by establishing a xenograft model involving HCC cells that overexpress miR-125a-5p to better understand how miR-125a-5p affects the development and progression of HCC.

It has been reported that silencing of NEAT1 can enhance HCC progression by inhibition of EGFR expression [24]. In keeping with this, the results presented here indicate that the expression of NEAT1 is abnormally high in HCC, and downregulation of NEAT1 promoted apoptosis in HEPG2 cells. In addition, downregulation of NEAT1 may inhibit angiogenesis. In human glioma, NEAT1 may, as a ceRNA of miR-194-5p, participate in the anti-angiogenic effect of isoliquiritigenin [36]. NEAT1 may regulate TGF-β by sponging miR-139-5p in HCC [37]. Yan et al. found that NEAT1 expression was significantly downregulated in cardiomyocytes after *in vivo* ischemia/reperfusion and *in vitro* H_2_O_2_ treatment. Further research has shown that NEAT1 can attenuate miR-125a-5p/BCL2L12 interaction in rat cardiomyocytes, thereby inhibiting H_2_O_2_-induced apoptosis [38]. Downregulation of NEAT1 promotes M2 polarization in mouse macrophages through the miR-125a-5p/TRAF6/TAK1 axis to alleviate LPS-induced inflammation [39]. The disease types, species, and downstream target proteins investigated in this study differ from those in the above studies. Our study found that NEAT1 was upregulated and miR-125a-5p was downregulated in HCC tissues. Further *in vitro* cell experiments showed that NEAT1 inhibition can promote apoptosis in HCC cells (HepG2) and inhibit angiogenesis in endothelial cells (HUVEC). NEAT1 may regulate VEGF expression through sponge adsorption of miR-125a-5p in HCC cells. The study is the first to report that NEAT1 may inhibit HCC by targeting miR-125a-5p/VEGF.

The VEGF receptor is a transmembrane tyrosine kinase receptor. It is widely distributed on the surface of endothelial cells, and binding of VEGF catalyzes the intraregional tyrosine kinase insertion region and activates the PI3K or MAPK pathway to induce a cascade of intracellular reactions that result in the proliferation and differentiation of cells [40,41]. In VEGF-induced tumor cells, the PI3K/AKT/mTOR pathway, which is the downstream pathway of VEGFR, can be activated to mediate the adhesion, proliferation, and migration of endothelial cells [42]. In addition, the ERK pathway is an important downstream pathway of VEGFR that mediates angiogenesis [43]. In the present study, we found that the angiogenesis-related downstream pathways of VEGFR, AKT/mTOR, and ERK were significantly activated in HCC tissues. In HepG2 cells, the AKT/mTOR and ERK pathways were significantly suppressed by miR-125a-5p, indicating that the antitumor properties of miR-125a-5p may be related to its inhibitory effects on the AKT/mTOR and ERK pathways. Furthermore, in the miR-125a-5p and VEGF co-incubation system, angiogenesis was enhanced and accompanied by activation of AKT/mTOR and ERK pathway.

Taken together, our data indicate that NEAT1 appears to promote angiogenesis by acting as a ceRNA of miR-125a-5p and by affecting the AKT/mTOR/ERK/VEGF signaling pathway.
